# Altered Gray Matter Volume in Patients With Type 1 Diabetes Mellitus

**DOI:** 10.3389/fendo.2020.00045

**Published:** 2020-02-13

**Authors:** Jia Liu, Wenliang Fan, Yuxi Jia, Xiaoyun Su, Wenjun Wu, Xi Long, Xin Sun, Jie Liu, Wengang Sun, Tianjing Zhang, Qiyong Gong, Haojun Shi, Qing Zhu, Jing Wang

**Affiliations:** ^1^Department of Radiology, Union Hospital, Tongji Medical College, Huazhong University of Science and Technology, Wuhan, China; ^2^Hubei Province Key Laboratory of Molecular Imaging, Wuhan, China; ^3^Philips Healthcare, Guangzhou, China; ^4^Department of Radiology, Huaxi MR Research Center, West China Hospital of Sichuan University, Chengdu, China; ^5^Department of Neurology, Tongji Medical College, Union Hospital, Huazhong University of Science and Technology, Wuhan, China

**Keywords:** gray matter, type 1 diabetes mellitus, seed-based d mapping, meta-analysis, voxel-based morphometry

## Abstract

**Background and Purpose:** Many imaging studies have reported structure alterations in patients with type 1 diabetes mellitus (T1DM) by using voxel-based morphometry (VBM). Nevertheless, the results reported were inconsistent and had not been reviewed quantitatively. Accordingly, the quantitative meta-analysis which including VBM studies of patients with T1DM was conducted.

**Materials and Methods:** The gray matter volume alterations in patients with T1DM was estimated by using the software seed-based d mapping. Meantime, the meta-regression was applied to detect the effects of some demographics and clinical characteristics.

**Results:** Six studies were finally included, which with 6 datasets comprising 414 T1DM patients and 216 healthy controls. The pooled meta-analyses detected that patients with T1DM showed robustly increased gray matter volume in the left dorsolateral superior frontal gyrus and middle frontal gyrus and a decreased gray matter volume in the right lingual gyrus, cerebellum, precuneus, the left inferior temporal gyrus, and middle temporal gyrus. The meta-regression showed that the mean age, the female patient's ratio, duration of illness and HbAlc% for T1DM patients were not linearly related with gray matter alterations.

**Conclusion:** This meta-analysis demonstrates that gray matter volume decreases in T1DM patients were mainly locates in the cortical regions and cerebellum.

## Introduction

Type 1 diabetes mellitus (T1DM) is a chronic metabolic disease which often occurs in children (1). During the past few years, the amount of T1DM patients has sharply increased in lots of countries (2). As a progressive disease, glucose dysregulation in T1DM can lead to severe complications, including neuropathies, which has been reported to increase the incidence of cognitive deficits and psychological dysfunction (3, 4). An important tool brain imaging is always being used to explore the mechanisms linking T1DM and cognitive dysfunction. Among many kinds of brain imaging modalities, Magnetic Resonance Imaging (MRI) is one of the tools that is commonly used in cognitive study.

Many previous studies have reported gray matter volume alterations in many different brain regions in patients with T1DM, which includes the superior frontal gyrus, middle frontal gyrus, middle temporal gyrus, thalamus and cerebellum (5–9). These changes are thought have some relations with the cognitive impairment in patients with T1DM. Nevertheless, based on the prior knowledge, some regions of interest in these studies were predefined, which may have resulted in potentially biased results. To overcome the technical limitations of region-of-interest, one method named voxel-based morphometry (VBM) could be selected, it is an automated whole-brain based analysis, which can unbiased investigate gray matter differences between patients and controls (10). Although most studies using VBM have observed gray matter alterations in T1DM patients, results were different. For example, a prior study found decreased gray matter volume in T1DM subjects compared with control subjects bilaterally in cerebellum, precuneus, cuneus, calcarine, lingual gyrus, and fusiform gyrus, and increased gray matter volume in T1DM subjects relative to control subjects in the left lateral prefrontal cortices, extending into the superior temporal gyrus, middle temporal gyrus, and insula (7). Another study reported that T1DM had decreased gray matter volume in the left middle temporal gyrus, right postcentral gyrus, and left triangular part of the frontal inferior gyrus. No brain regions presenting increased GMV in the T1DM subjects in this study (9). Many variables such sample size, protocols used and the patient's clinical and demographic characteristics may account for the inconsistencies. Therefore, we intent to conduct a meta-analysis in patients with T1DM to detect consistent results from studies using VBM.

A new coordinate-based meta-analytic method named seed-based d mapping (SDM) has been used for meta-analyzing studies about brain structure or activity differences. Different kinds of neuroimaging techniques such as fMRI (11), VBM (12), or DTI (13) were being used in these studies. Many diseases has been analyzed by using SDM, such as depression (11), posttraumatic stress disorder (14), and dementia (15). Many useful features of activation likelihood estimation and multilevel kernel density analysis were combined in the SDM. It also superior than these methods in some respects. For instance, differences including positive and negative can be combined in one map, thus a particular voxel from may be significant in opposite directions can be prevented (16). Meanwhile, the SDM also contains some extra analyses which are using to estimate the robustness and heterogeneity of results. These analyses include jack-knife, subgroup and meta-regression analyses (17). However, the meta-analysis includes VBM studies comparing patients with T1DM and healthy controls has not ever been analyzed by SDM. Therefore, we intent to use SDM to detect gray matter alterations between T1DM patients and healthy controls by quantitatively review the previous studies using VBM about T1DM.

## Methods

### Inclusion of Studies

Guideline PRISMA was used in our meta-analysis (18). The databases we used in our study were PubMed, Web of Science and Medline, and the time of publication was from January 1947 to June 2019. The keywords included “diabetes” or “diabetes mellitus” plus “voxel-based morphometry,” “voxel-based,” “voxel-wise,” “morphometry,” or “VBM.” Additionally, we also manually checked some review articles and the identified articles' references. The inclusion criteria were: (1) the gray matter differences between patients with T1DM and healthy controls were compared at the whole-brain level (2) the results of comparisons were demonstrated in a stereotactic space in three coordinates (x, y, and z). Once several independent samples were contained in one study, their coordinates were included separately. And the studies were excluded if they use ROI or seed voxel-based analysis. If some articles lack coordinates, we would contact the author to decrease the likely of a biased sample set.

### Quality Assessment

The checklist with 13 point was used to examine the studies qualities we included. Which focus on some basic information of the study samples and imaging-specific methodology. The checklist was made in accordance with the studies published before (12, 19). The contents of the checklist were included in the Table S1. Despite the checklist was not used to assess, it can provide an objective indication of the rigor of each study. Two authors reviewed the studies independently, and then got completeness rating. Once rating disagreement came up, the paper would be discussed until the same score was achieved. The scores are demonstrated in Table 1.

**Table 1:** Demographic and clinical characteristics of the participants in the 6 voxel-based morphometry studies included in the meta-analysis.

	**Patients with type 1 diabetes**						**Healthy controls**				
**References**	**No (%female)**	**Mean age, year**	**Illness Duration, year**	**Age of onset**	**HbAlc%**	**Cormobidity (number of patients)**	**No (%female)**	**Mean age, year**	**MRI**	**Software**	**Quality scores**
Musen et al. (5)	82 (60.0)	32.6	20.3	NA	7.8	Retinopathy (51)	36 (55.6)	31.3	1.5T	SPM99	11.5
Wessels et al. (20)	31 (61.0)	40.6	26.3	14.2	8.0	Diabetic proliferative retinopathy (13); 4 DRP patients have nephropathy; nephropathy and neuropathy (2)	21 (66.7)	36.3	1.5T	SPM2	11.5
Perantie et al. (21)	108 (42.3)	12.6	5.7	6.9	8.4	NA	51 (49)	12.3	1.5T	SPM5	12.0
Kaufmann et al. (6)	30 (53.3)	14.3	5.6	7.65	8.3	NA	19 (52.6)	13.0	1.5T	SPM8	12.5
Marzelli et al. (7)	142 (46.5)	7.0	2.9	4.1	7.9	NA	68 (48.5)	7.0	3T	SPM8	12.5
Liu et al.	21 (62.0)	9.29	0.56	8.73	11.15	NA	21 (62%)	9.38	3T	FSL	12.5

*DRP, diabetic proliferative retinopathy; yr, year*.

### Voxel-Wise Meta-Analysis

SDM was used to detect gray matter differences between patients with T1DM and healthy controls (https://www.sdmproject.com/). First, a pooled meta-analysis of the included studies was performed. Some previous articles have described the details about SDM method (17, 22). We just briefly described it here. Firstly, peak coordinates we need were selected. And each study included should using the same statistical threshold at the whole brain, which can avoid potential bais. Secondly, a Gaussian Kernel was used to recreate a standard Talairach map for each study of the gray matter differences. The peak *t*-value was converted to Hedges effect size, which mean to recreate the peak coordinates. Then, a non-normalized Gaussian kernel was used to arrange larger values to the voxels closer to peaks. The differences for null findings was that voxels in the effect size map were arranged null effect size while the same effect size was recreated. The random-effects meta-analytic also included the null effect size as other effect sizes. Thirdly, the voxel-wise calculation was done to get the mean map, and the calculation was weighted by the square root of the sample size of each study. Therefore, the larger studies would contribute more. At last, a randomizations tests was done to evaluate statistical significance, and null distribution was created by which can directly get *p*-values. Default SDM kernel size and thresholds were used (full-width at half-maximum = 20 mm, voxel *p* = 0.005, peak height *Z* = 1, cluster extent = 10 voxels) (17). Besides, the jack-knife sensitivity analysis was used to evaluate the reliability of the results. For instance, the data sets were analyzed six times in the pooled meta-analysis, with one data set discarded every time. The principal of the test is to detect if a significant brain region is still significant in all or the majority of the studies, which suggested the result is highly replicable.

### Meta-Regression Analysis

Some sociodemographic and clinical variables showed in Table 1 were calculated by simple linear regression to detect the potential effects, which was weighted by the square root of the sample size and restricted to predict only possible SDM values (i.e., from −1 to 1) in the observed range of values of the variable (17). Each variable can get a map of regression slope. As in published meta-analysis, we also decreased the probability threshold to 0.00005 to minimize the finding of spurious associations, required abnormalities to be detected both in the slope and in 1 of the extremes of the regressor, and discarded findings in regions other than those detected in the main analyses. Finally, if the result was driven by too small studies, the regression plots would be discarded (22).

## Results

### Studies Included in the Meta-Analyses

**Figure 1:**
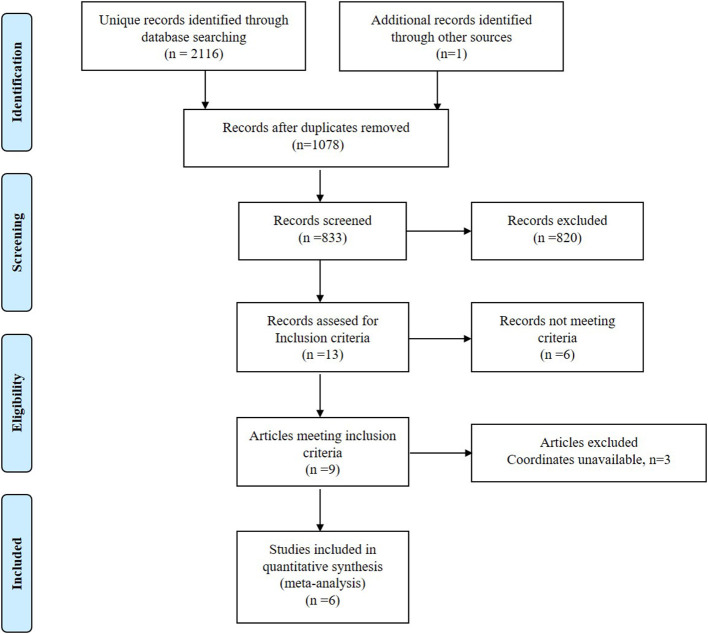
Meta-analysis of voxel-based morphometry studies in patients with type 1 diabetes mellitus.

The studies identification and attrition are demonstrated in Figure 1. A total of 2,116 studies were identified in our study, and 5 studies met the inclusion criteria. Additionally, one study was found in the reference lists. Therefore, 6 VBM studies were finally included. Some data including clinical and demographic data can be detected in Table 1. The software that each study used to do VBM were Table 1. Five of the studies used SPM and one of the studies used FSL. Each study used the VBM toolbox in the software. The T1DM and control groups had no significant difference in age or sex in each study. However, significant difference exists between the two groups when considering the whole data set (*P* > 0.05). Besides, the mean age was 19.40 years in the T1DM patients vs. 18.21 years in the healthy controls, and women there were 209 (50.00%) in the T1DM vs. 115 (53.24%) in the control.

### Meta-Analysis of All Studies

**Figure 2:**
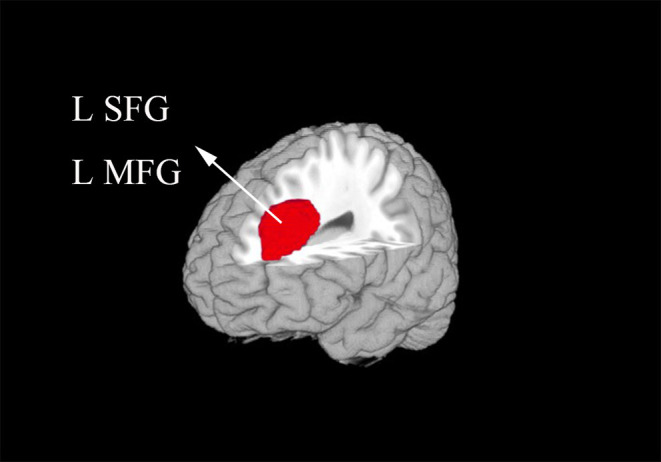
The areas of increased gray matter volumes in patients with type 1 diabetes mellitus (T1DM) compared with Healthy controls in the meta-analysis. Gray matter volume changes in patients with T1DM are displayed on a 3-dimensionally rendered brain, with part of the left or right hemisphere removed. L, left; MFG, middle frontal gyrus; SFG, superior frontal gyrus.

**Figure 3:**
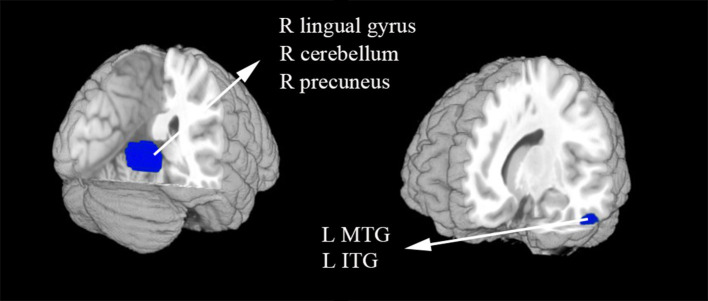
The areas of decreased gray matter volumes in patients with type 1 diabetes mellitus (T1DM) compared with Healthy controls in the meta-analysis. Gray matter volume changes in patients with T1DM are displayed on a 3-dimensionally rendered brain, with part of the left or right hemisphere removed. ITG, inferior temporal gyrus; L, left; MTG, middle temporal gyrus; R, right.

In the meta-analysis, patients with T1DM showed increased gray matter volume in the left dorsolateral superior frontal gyrus (SFG) and middle frontal gyrus (MFG). And decreased gray matter volume in the right lingual gyrus, cerebellum, precuneus, the left inferior temporal gyrus (ITG), and middle temporal gyrus (MTG) (Table 2 and Figures 2, 3).

**Table 2:** Regional differences in gray matter volume between patients with type 1 diabetes and healthy controls in the pooled meta-analysis (voxel-wise *p* < 0.005 and full-width at half-maximum 20 mm).

	**Maximum**			**Clusters**	
**Brain regions**	**MNI coordinates x, y, z**	**SDM value**	***p*-value**	**No. voxel**	**Breakdown (No. of voxels)**
**DIABETES>CONTROL**					
L superior frontal gyrus, dorsolateral	−24, 34, 36	1.547	0.000004292	947	L superior frontal gyrus, dorsolateral, B9, 46, 32 (193) L middle frontal gyrus, BA 9, 45, 46 (754)
**DIABETES < CONTROL**					
R lingual gyrus	10, −50, 2	−1.547	0.000435233	424	R lingual gyrus, BA17, 18, 19, 27, 29, 30, 37 (337) R cerebellum, hemispheric lobule IV/V, BA18, 30 (55) R precuneus, BA17, 27, 29, 30, 37 (24) R parahippocampal gyrus, BA27, 30 (8)
L middle temporal gyrus	−56, −8, −26	−1.159	0.003499866	49	L inferior temporal gyrus, BA20, 21 (28) L middle temporal gyrus, BA20, 21 (21)

*BA, Brodmann area; L, Left; MNI, Montreal Neurological Institute; R, right; SDM, signed differential mapping*.

### Reliability Analysis

The Table 3 showed the whole-brain jack-knife sensitivity analysis of the meta-analysis. Which found that the gray matter volume increased significantly in the left dorsolateral SFG and MFG and decreased significantly in the right lingual gyrus, cerebellum, precuneus, and the left ITG in all but one combination of the data sets. The decreased gray matter volume in the left MTG was significant in all but two combination of the data sets.

**Table 3:** Sensitivity analyses of voxel-based morphometry studies of gray matter in patients with type 1 diabetes in the meta-analysis.

	**Decreased gray matter**		**Increased gray matter**				
**Discarded study**	**L**	**L**	**R**	**R**	**R**	**L**	**L**
	**SFG**	**MFG**	**lingual gyrus**	**cerebellum**	**precuneus**	**MTG**	**ITG**
Musen et al. (5)	Y^&^	Y	Y	Y	Y	Y	Y
Wessels et al. (20)	Y	Y	Y	Y	Y	N	Y
Perantie et al. (21)	N^$^	N	N	N	N	Y	Y
Kaufmann et al. (6)	Y	Y	Y	Y	Y	N	N
Marzelli et al. (7)	Y	Y	Y	Y	Y	Y	Y
Liu et al.	Y	Y	Y	Y	Y	Y	Y

*ITG, inferior temporal gyrus; L, left; MFG, middle frontal gyrus; MTG, middle temporal gyrus; N, no; R, right; SFG, superior frontal gyrus; Y, yes*.

& *Remained significantly increased/decreased after exclusion of the study in the jack-knife analysis*.

$ *No longer significantly increased/decreased after exclusion of the study in the jack-knife analysis*.

### Meta-Regression

The information on the mean age, the percentage of female patients with T1MD, illness duration and HbAlc% were evaluated by meta-regression in the patient group. This information can get from all 414 participants in the 6 studies. There variables were finally found not linearly associated with gray matter volume alterations.

## Discussion

As far as we know, our study is the firstly using SDM to pool VBM studies for a meta-analysis, which mean to find gray matter differences between patients with T1DM and healthy controls. The results identified gray matter changes mainly located in the cortical regions. And part of the cerebellum.

Among the included 6 VBM studies, two studies found no significant differences between T1DM and healthy controls, other 4 studies reported decreased gray matter volume in T1DM relative to healthy controls, and only 1 study showed increased gray matter volume in T1DM compared with healthy controls. Most of the studies reported decreased gray matter volume in the temporal gyrus in T1DM. Decreased gray matter volume of some brain regions in parietal and occipital lobe were also detected. The decreased gray matter in the frontal gyrus is rarely reported when compared T1DM with healthy controls.

Both increased and decreased gray matter volume of the brain regions were detected in our study. One executive task-based fMRI study of children with T1DM found higher activation in executive control regions and decreased suppression of activation in the posterior node of the default mode network. And the study concluded that increased recruitment of executive control areas in pediatric T1DM may lead to offset diabetes-related damage of default mode network, which may facilitate cognitive and behavioral performance levels that are similar to that of non-diabetic controls (23). The relationship between functional and structural alterations in the patients with T1DM may need to be further confirmed in the future.

Our study identified increased gray matter volume in the left dorsolateral SFG and MFG, which are parts of frontal cortex. The frontal cortex is an important part that associated with emotional processing and cognitive regulation, which includes attention and behavioral control (24). As we can see from the included studies, the result of abnormal gray matter of frontal regions was mainly contributed by the studies which included adults. These patients were with long illness duration and chronic hyperglycemia. Previous studies had shown that chronic hyperglycemia seems impact more on poor cognitive function for adults with T1DM (25, 26). A prior fMRI study showed that middle-aged patients with T1DM showed higher brain activation compared with persons without T1DM in the frontal cortex while performing a psychomotor speed task (27). The increased gray matter volume in clinical has not studies well. One explanation is that use-dependent brain expansion may occur to support heavy reliance on particular brain regions (28), which need to be confirmed by further studies.

Our meta-analysis found significantly lower gray matter volume in the right lingual gyrus, precuneus, and the left ITG and MTG in T1DM. More and more evidence that showed that lowered cognitive performance in patients with T1DM is related to chronic hyperglycaemia (29, 30). Hyperglycaemia may result in an accumulation of potentially toxic glucose metabolites, oxidative stress, accelerated formation of advanced glycation end-products and microvascular changes in the brain (31). Hyperglycaemia-induced alterations may lead to accelerated aging of the brain, such as cortical atrophy of temporal, parietal and occipital regions. A whole brain analysis found both decreased gray and white matter volume in T1DM relative to healthy control subjects with a higher brain perfusion (32). One prior study also showed lower volume in the temporal-parietal-occipital cortex in the T1DM patients compared with controls by means of regions-of-interest (33). A study about disrupted gray matter network properties found that patients without proliferative retinopathy showed alter local clustering and path length in the temporal and occipital gyrus (34). Lower axial diffusivity in the temporal and parietal regions in children with T1DM also reported in a previous study (35). Another DTI study found a negative correlation between the ADC values of the parietal white matter, and indicate that brain damage may have begun at the cellular level in the preliminary stage of T1DM and neurocognitive impairments can't be avoided (36). Meanwhile, one DTI study found the fractional anisotropy was decreased in posterior white matter tracts, which have high connectivity with lots of posterior cortical regions, including the cuneus, precuneus, fusiform, and posterior parietal cortical regions (37). According to the prior studies, we conjecture that loss of gray matter in the occipital and temporal gyrus may also owe to early retinal alterations that happen in type 1 diabetic patients (even in those without clinically evident diabetic retinopathy) (5, 20). As the recurrent episodes of severe hypoglycaemia, the influence of hypoglycemia to the brain gray matter volume cannot be ignored. Bednarik et al. reported that T1DM and damaged awareness of hypoglycemia were correlated with reduced gray matter volumes in the parietal and temporal lobe (38).

Furthermore, the patients with T1DM exhibited lower gray matter volume within the cerebellum. Previous study have speculated that the cerebellum may operate as a type of internal timing system (39), and it is associated with executive control and inhibition of distractors (40). Additionally, one study showed that the structure of cerebellum can be mostly influenced during neurodevelopment in children with T1DM (7). This result could account for the gray matter volume differences between the groups in our study. Besides, elevated HbA1c at diagnosis was found correlated with decreased volume in cerebellar white matter (33). Except for structure differences, some studies also found activation differences between T1DM and healthy controls in cerebellum (41, 42).

Our study has several limitations. Firstly, the sample size was small, which may limit the power of our analyses. Secondly, the data acquisition, analysis techniques, patient characteristics, and clinical variables were varied in the studies. For instance, some patients with T1DM in our study came up recurrent episodes of severe hypoglycaemia beside chronic hyperglycaemia, the unstable blood glucose may affect brain structure. However, we cannot investigate most of these variables in our study as we can't get the detailed information to do a subgroup or meta-regression analysis (22). Thirdly, our study performed the meta-analysis with a combination of children and middle-aged individuals, but the number of included studies was in sufficient for a subgroup analysis. Which can be done in the future if more studies included. Fourth, some limitations about the VBM method still exist. From the Table 1 we can observe that 4 versions of SPM and 1 FSL were used in these studies. Different version of SPM may produce different results due to different pre-processing procedures. It's better to do a subgroup analysis or perform a meta-analysis by including studies using the same version of software if the number of the studies is enough. Although, the VBM has been well-adapted for coordinate-based meta-analyses. Voxel-based morphometry analyses may over estimate group differences in areas of high anatomic differences (43, 44) and may be biased toward detecting highly localized group differences and against detecting ones that are spatially more diverse. Finally, the using of coordinate-based methods may result in less accurate results as it was based on summarized statistical brain maps (45).

## Conclusion

The results of our meta-analysis suggest that T1DM patients have significantly and stable decreased gray matter volume in the cortical regions and cerebellum. This study may support the opinion that T1DM could lead to subtle diabetic brain structural changes, which can be associated with cognitive deficits in T1DM patients. Further study may need to investigate the dynamic brain structure alterations in patients with T1DM and its relationship with cognition, which can help us better understand the results.

## Data Availability Statement

The datasets generated for this study are available on request to the corresponding author.

## Author Contributions

JW, QZ, and HS contributed to the conception of the study. JiaL, WF, YJ, XSu, WW, XL, XSun, JieL, WS, and TZ contributed significantly to analysis and manuscript preparation. JiaL performed the data analyses and wrote the manuscript. QG contributed to the interpretation and discussion of the results of the analysis.

### Conflict of Interest

TZ was employed by the company Philips Healthcare. The remaining authors declare that the research was conducted in the absence of any commercial or financial relationships that could be construed as a potential conflict of interest.
