# Corrigendum: Altered Gray Matter Volume in Patients With Type 1 Diabetes Mellitus

**DOI:** 10.3389/fendo.2020.00604

**Published:** 2020-08-28

**Authors:** Jia Liu, Wenliang Fan, Yuxi Jia, Xiaoyun Su, Wenjun Wu, Xi Long, Xin Sun, Jie Liu, Wengang Sun, Tianjing Zhang, Qiyong Gong, Haojun Shi, Qing Zhu, Jing Wang

**Affiliations:** ^1^Department of Radiology, Union Hospital, Tongji Medical College, Huazhong University of Science and Technology, Wuhan, China; ^2^Hubei Province Key Laboratory of Molecular Imaging, Wuhan, China; ^3^Philips Healthcare, Guangzhou, China; ^4^Department of Radiology, Huaxi MR Research Center, West China Hospital of Sichuan University, Chengdu, China; ^5^Department of Neurology, Tongji Medical College, Union Hospital, Huazhong University of Science and Technology, Wuhan, China

**Keywords:** gray matter, type 1 diabetes mellitus, seed-based d mapping, meta-analysis, voxel-based morphometry

In the published article, there was an error in affiliations 1 and 5. Instead of “**Department of Radiology, Tongji Medical College, Union Hospital, Huazhong University of Science and Technology, Wuhan, China**,” it should be “**Department of Radiology, Union Hospital, Tongji Medical College, Huazhong University of Science and Technology, Wuhan, China**.”

The error in affiliation 5 should be instead of “**Department of Neurology, Tongji Medical College, Union Hospital, Huazhong University of Science and Technology, Wuhan, China**,” it should be “**Department of Neurology, Union Hospital, Tongji Medical College, Huazhong University of Science and Technology, Wuhan, China**.”

The authors apologize for these errors and state that this does not change the scientific conclusions of the article in any way. The original article has been updated.

